# Supplementary material for: Multiple signals evoked by unisensory stimulation converge onto cerebellar granule and Purkinje cells in mice
Source: Commun Biol. 2020 Jul 15;3:381. doi: 10.1038/s42003-020-1110-2 (PMC7363865; doi:10.1038/s42003-020-1110-2)
Supplement: Supplementary file 1 — Supplementary Information [file 42003_2020_1110_MOESM1_ESM.pdf]

Supplementary Figures

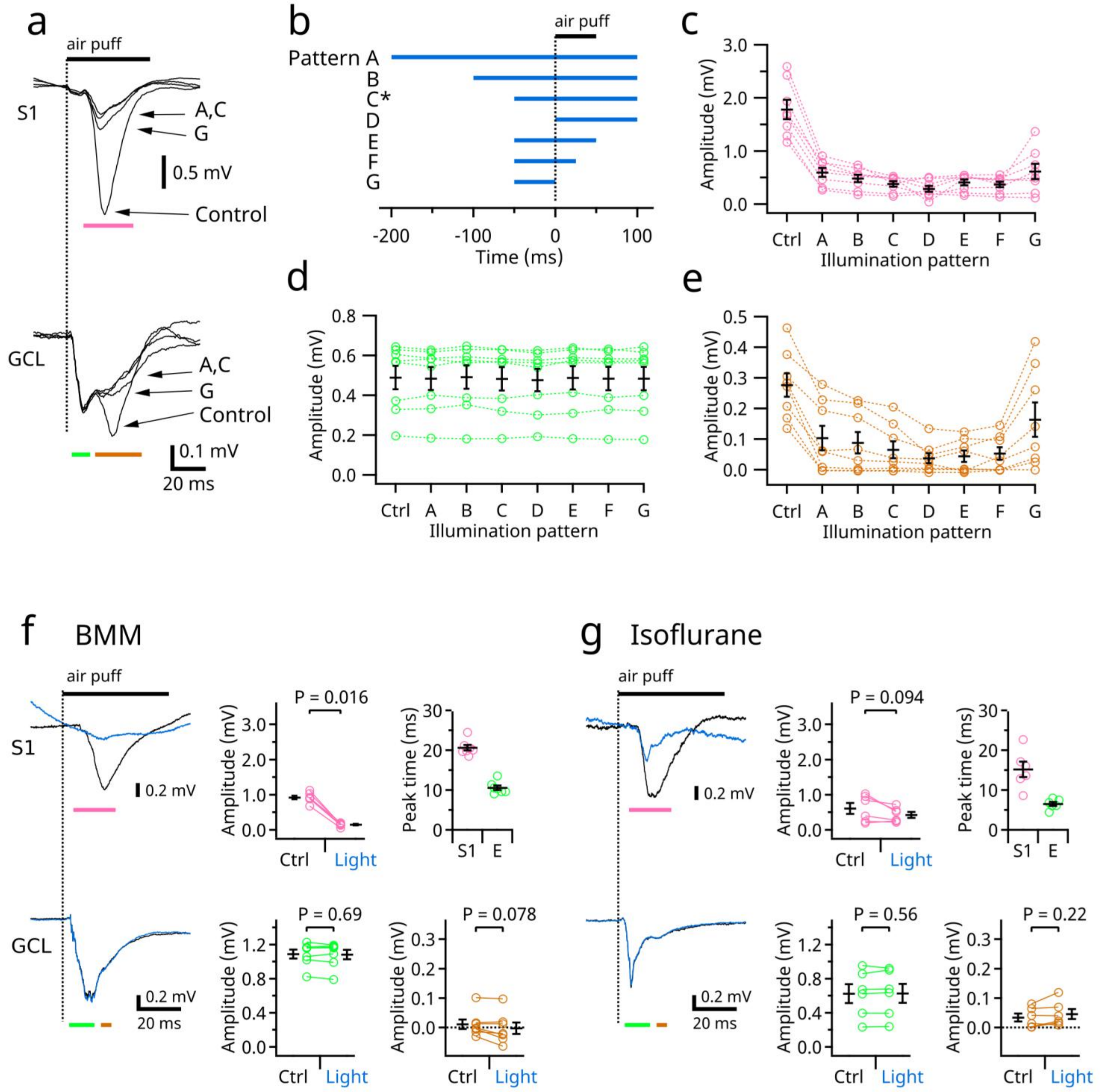

Supplementary Fig. 1 | Exploration of temporal patterns of S1 photoinhibition and effects of anesthetics. a, Representative recordings of field potential in S1 (top) and cerebellar GCL (bottom). The averages from 20 traces in each stimulation pattern are shown. **b**, Temporal patterns of the light illumination. The patterns B, D, E, and F are omitted in **a** for clarity. Black bar indicates the duration of air puff, whose onset is indicated by the vertical dotted line. **c**–**e**, The peak amplitudes of S1 field potentials (**c**) and the early (**d**) and the late (**e**) components of cerebellar GCL field potentials (n = 8). **f**, The same experiments as described for Fig. 2c,d, but the mice were anesthetized with a mixture of medetomidine (0.3 mg/kg), midazolam (4 mg/kg), and butorphanol (5 mg/kg) (BMM). **g**, The same as in **f**, but the mice were anesthetized with isoflurane (2%). Means ± SEMs are presented as black lines and bars, respectively.

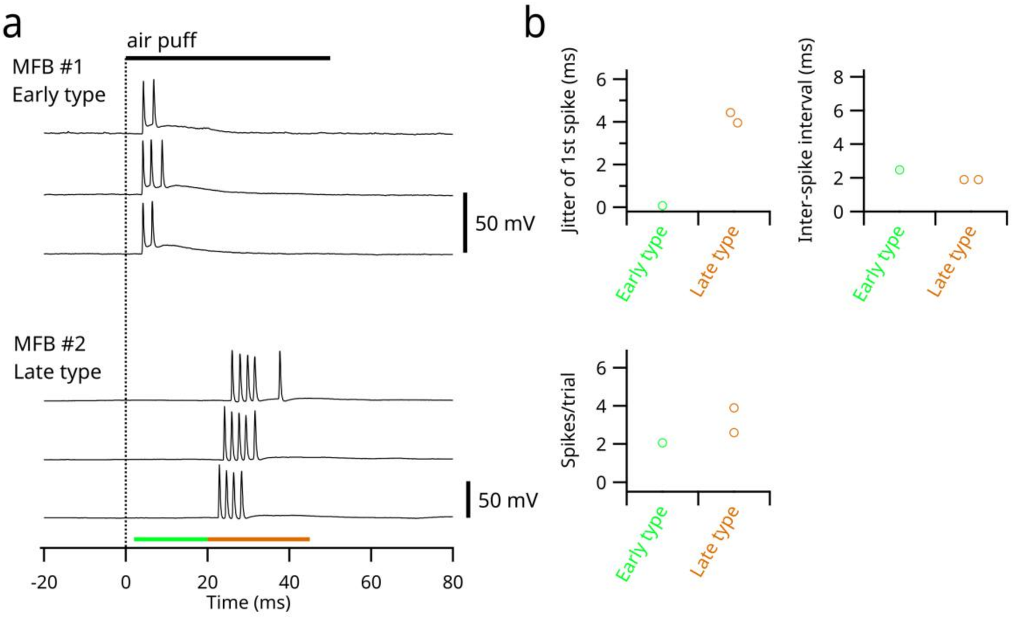

Supplementary Fig. 2 | Sensory responses in putative mossy fiber boutons. a, Whole-cell current clamp recordings from putative mossy fiber boutons (MFB; *n* = 3). MFB #1 was from a VGAT-ChR2 mouse, whereas MFB #2 was from an Aldoc-Venus mouse. Firing of MFB #1 was not affected by photoinhibition of S1 (not displayed). Three sample traces from each MFB are shown. MFBs were categorized as the early or the late type on the basis of their timing of firing. **b**, Spike patterns were analyzed in the same manner as described for Fig 3c,d,f.

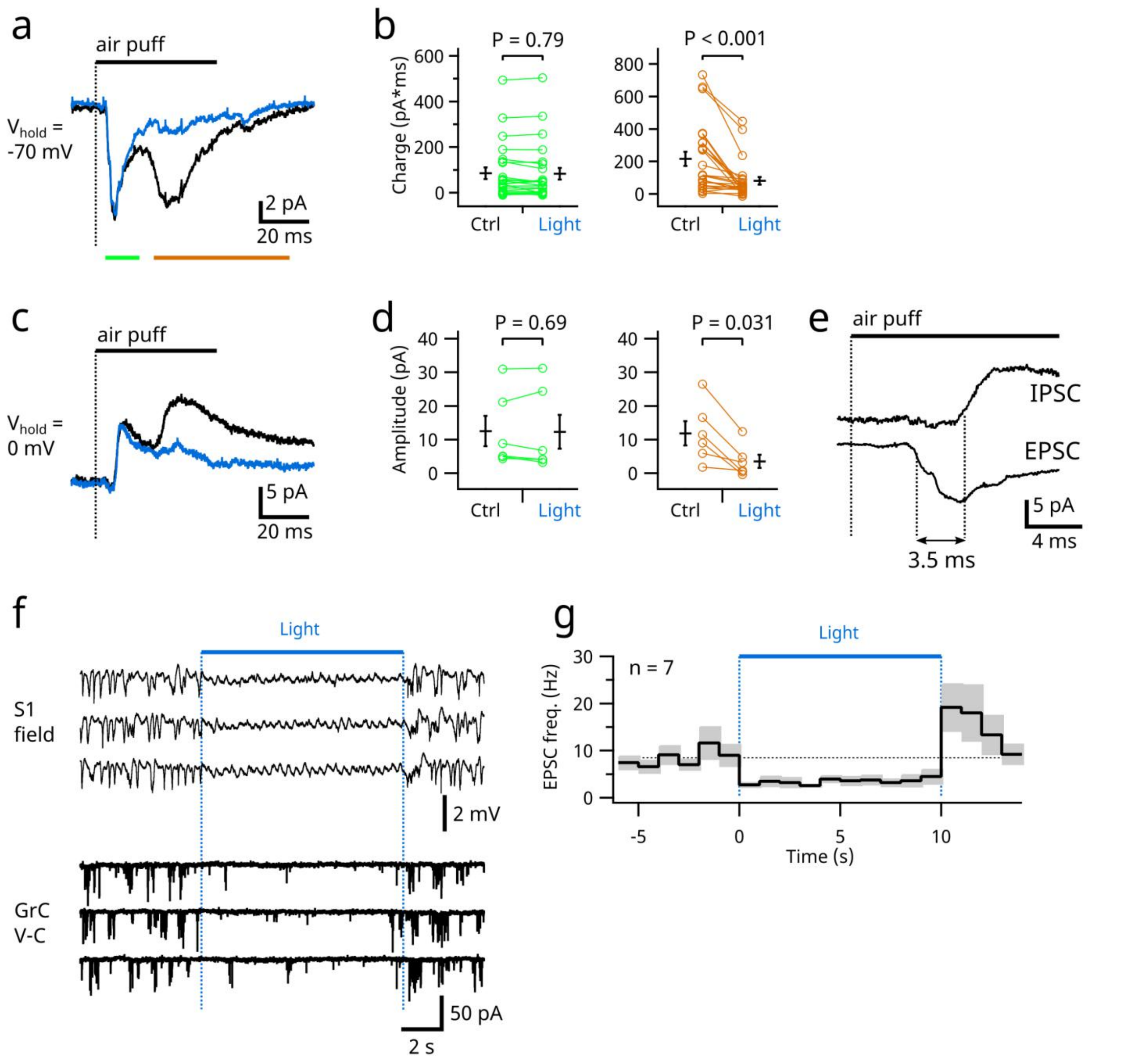

Supplementary Fig. 3 | Extra data from whole-cell voltage clamp experiments. a, Average traces of EPSCs in all 24 granule cells presented in Fig. 3. **b**, Synaptic charges (integral of the synaptic current, *n* = 24) measured for the time periods indicated in **a**. All types of granule cells, including cells lacking a late component, were included. The synaptic charges during the late phase (right, brown) may contain the tails of the early components (left, green). The synaptic charge, which can be measured without event detection, is an index related to both the number of events (Figure 3f) and the size of individual EPSCs (Figure 3e). **c**, Average traces of IPSCs from six granule cells voltage clamped at 0 mV (the reversal potential for the glutamatergic current). **d**, The late component of IPSCs was significantly suppressed (*n* = 6). **e**, Comparison of onsets of an EPSC and IPSC. Control traces in **a** and **c** are shown together. The onset (20% rising point) of the averaged traces had a 3.5 ms difference, reflecting one additional synaptic delay in the mossy fiber-Golgi cell-granule cell pathway. **f**, Representative recordings; simultaneous field potential recordings from S1 (top) and whole-cell voltage clamp recording from a granule cell (bottom). Long (10 s) blue light applications were repeated five times, but three trials are displayed. **g**, Frequencies of spontaneous EPSCs (average from seven granule cells) are displayed. Shading indicates SEMs. Means ± SEMs are presented as black lines and bars, respectively.

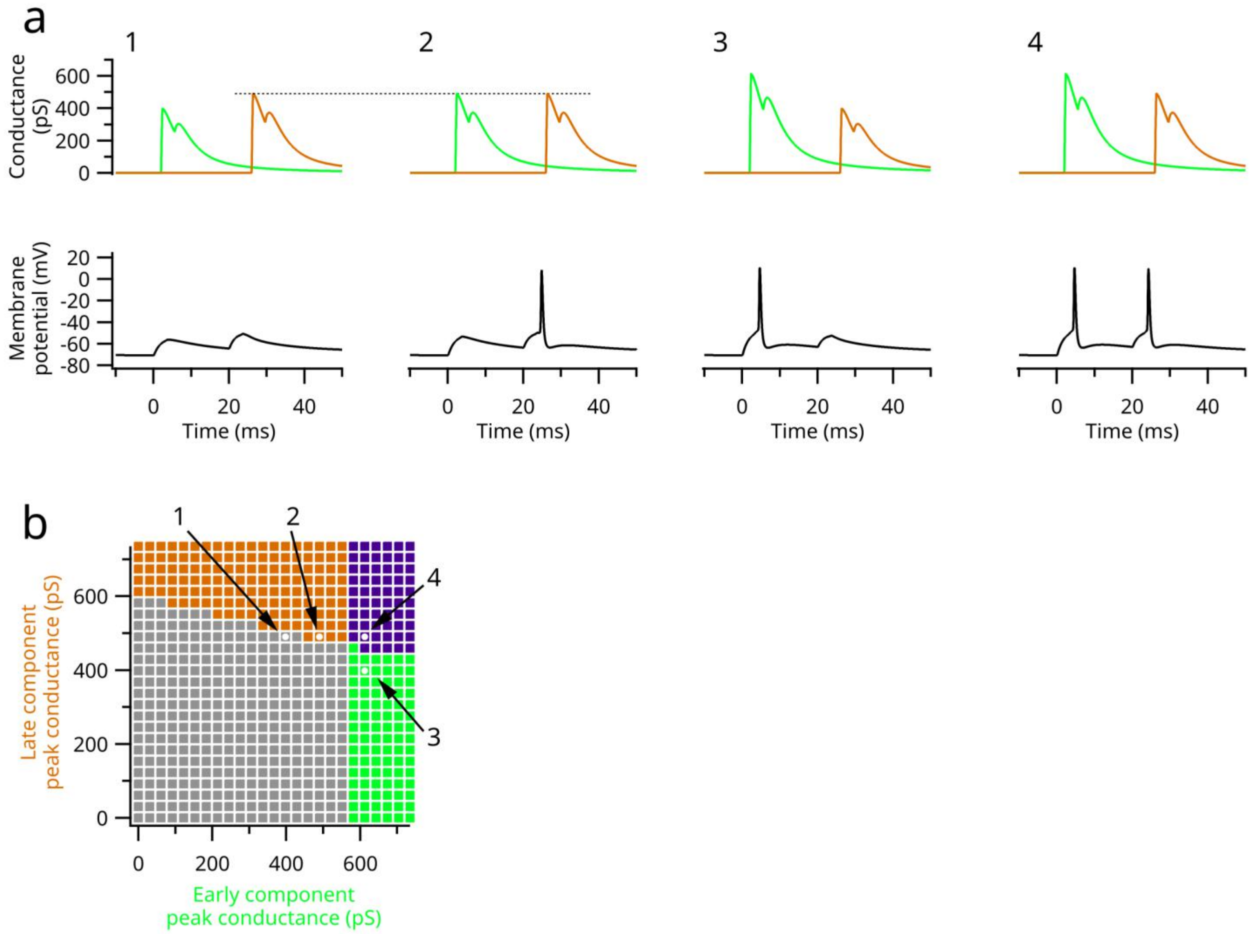

Supplementary Fig. 4 | Computer simulation of action potential generation in granule cells. **a**, Four representative patterns of simulation. Top, AMPA-type synaptic conductance. Bottom, resulting membrane potential at the soma. A realistic model of a granule cell was used as published by Diwakar et al.[1] for the NEURON simulation environment [2] without modifications except for AMPA and NMDA conductances, which were systematically changed while the NMDA/AMPA ratio was kept constant (0.2)[3,4]. Excitatory synaptic conductances, each of which consists of two synaptic events with a 3 ms interval, were applied to two dendrites with a separation of 20 ms. Inhibitory synaptic conductances, each of which consists of a single synaptic event with a 3.5 ms delay from the onset of excitatory synaptic conductance, were applied with a separation of 20 ms and a constant maximal conductance (756.35 pS). **b**, Overall profile of spiking patterns triggered by various synaptic conductances. A plot resembling that in Fig. 3b, but where the colors indicate the resulting spike patterns. White dots indicate the cases presented in **a**. This model contains the following elements that can potentially counteract the facilitation by temporal summation: (1) a decreased driving force of excitatory synaptic current by depolarization, (2) an increased driving force of inhibitory synaptic current by depolarization, (3) Na^+ channel inactivation by a prolonged depolarization, and (4) an increased dendritic leak current via Ca^{2+}-activated K channels activated by Ca^{2+} influx through NMDA receptors.

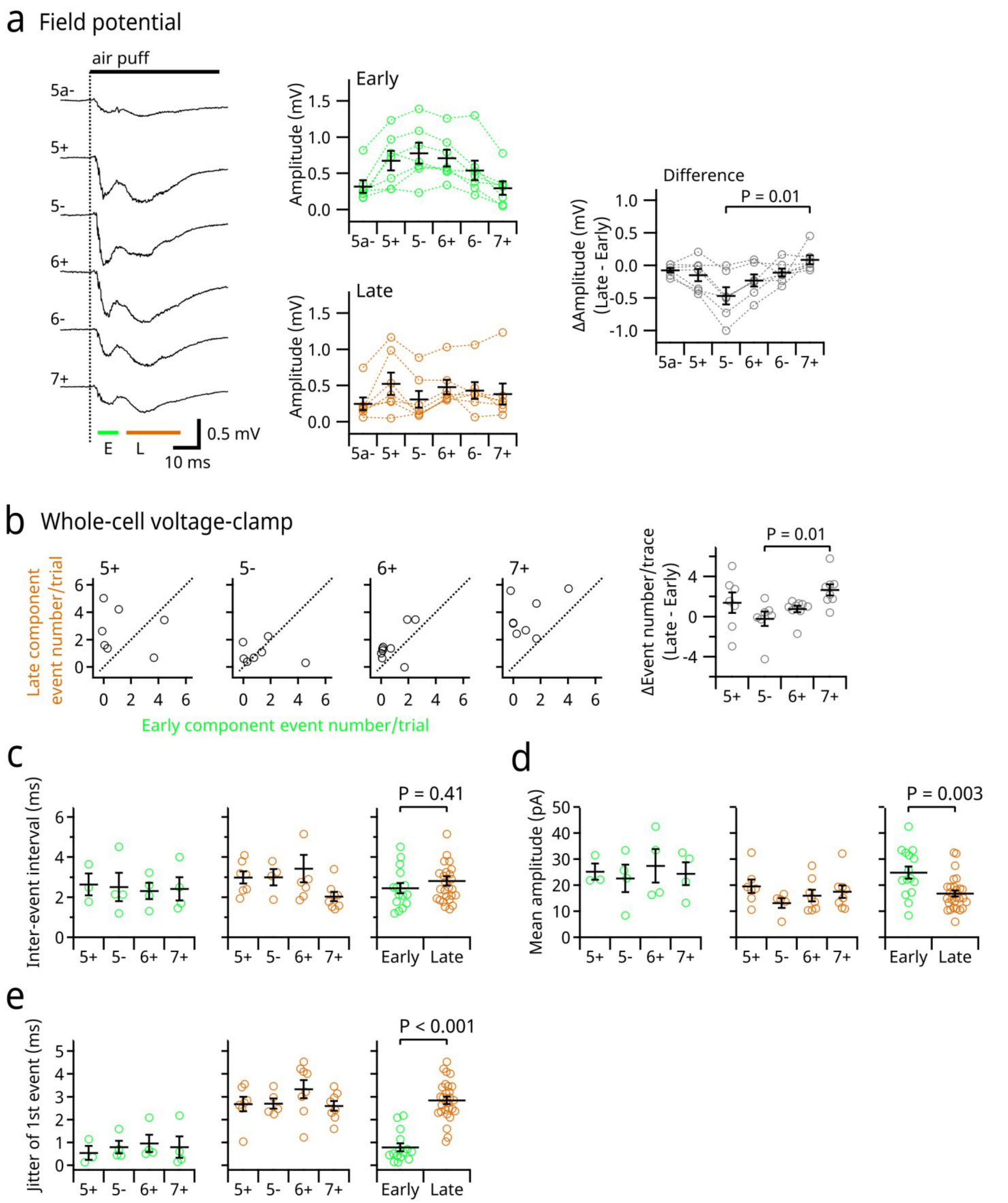

Supplementary Fig. 5 | Extra data from experiments using Aldoc-Venus mice. a, Left, representative field potential recordings from six different bands in crus II. Center, pooled data from seven mice. The amplitudes of early (top) and late (bottom) responses were measured during the times indicated by the colored bars in the left panel. Right, the differences of amplitudes between the early and late components in each mouse are plotted. In terms of the balance, the 7+ band had significantly more weight in the late component than the 5- band (pairwise multiple comparison). The other 14 pairwise comparisons did not have significant differences. **b**, Left, the numbers of evoked EPSC events in the early and the late phases were compared in individual bands. The same data as in Fig. 5c were used. Dotted lines indicate unity. Right, the

differences of event numbers/trial between the early and the late components in each granule cell are plotted. In terms of the balance, the granule cells in the 7+ band had significantly more weight in the late component than those in the 5- band (pairwise multiple comparison). The other 5 pairwise comparisons did not have significant differences. **c**–**e**, The median interevent intervals (**c**), the mean amplitudes (**d**), and the jitter (the standard deviation of the timing) (**e**) of EPSCs in various bands in the early (left) and the late (right) phases. The right panels show pooled data from all bands. Means ± SEMs are presented as black lines and bars, respectively.

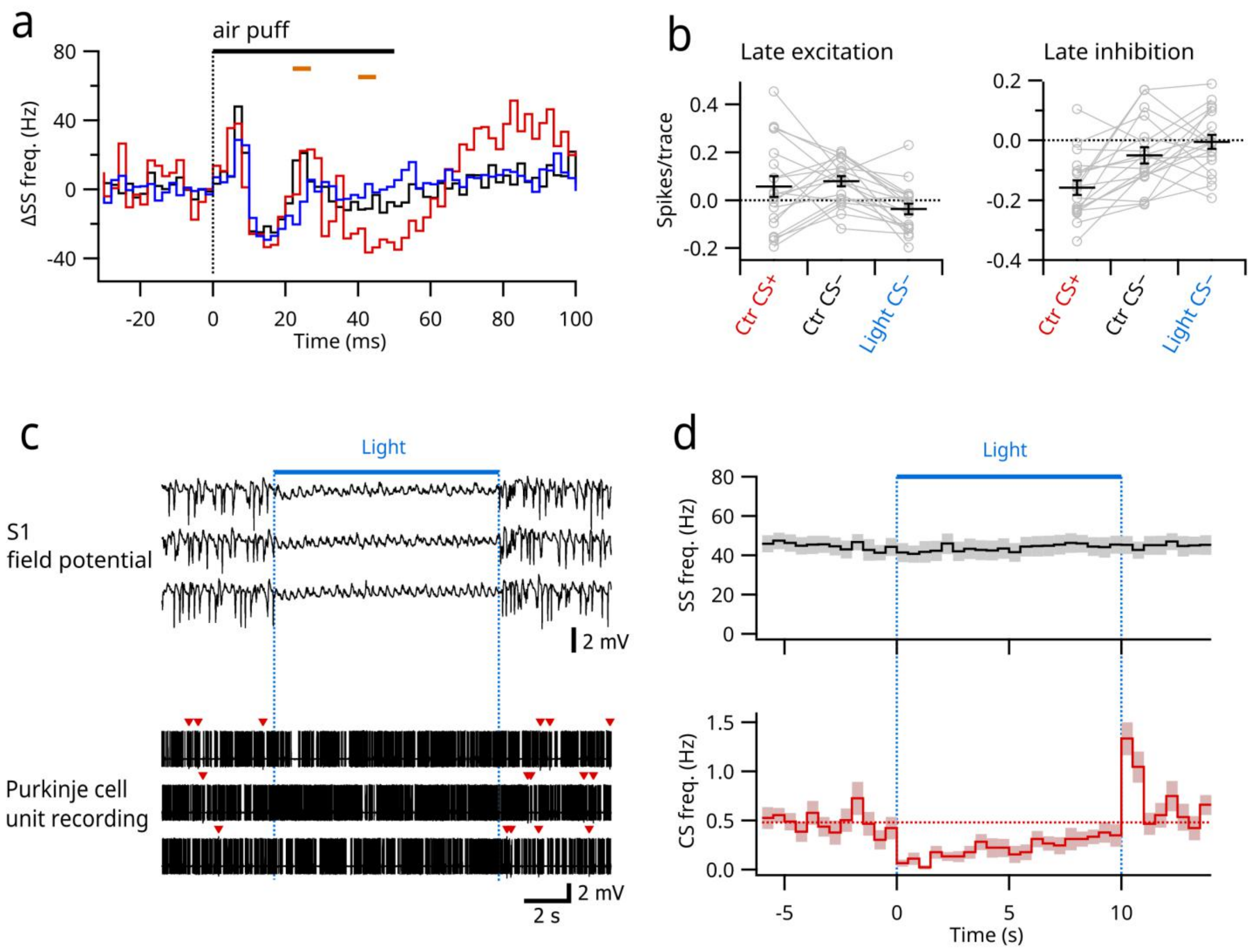

Supplementary Fig. 6 | Detailed analysis of the interaction of SS and CS and effects of S1 photoinhibition on spontaneous SS and CS in Purkinje cells. **a**, Average time histogram (2 ms bin, *n* = 19) of SS from trials with CS responses (red) and without CS responses (black) under the control condition and those without CS responses during photoinhibition of S1 (blue) are shown. In S1 photoinhibition, trials with CS responses were rare and therefore omitted in this analysis. **b**, SS numbers were measured during the times indicated by bars in **a**, the same timings as in Fig. 6d. **c**, Representative recordings; simultaneous field potential recordings from S1 (top) and unit recordings from a Purkinje cell (bottom). Long (10 s) blue light applications were repeated six times, but three trials are displayed. Red arrowheads indicate CS. **d**, Averages from 18 Purkinje cells. Spontaneous frequencies of SS (top) and CS (bottom) are displayed. Shading indicates SEMs. Means ± SEM are presented as black lines and bars, respectively.

Supplementary References

1. Diwakar, S., Magistretti, J., Goldfarb, M., Naldi, G. & D'Angelo, E. Axonal Na+ channels ensure fast spike activation and back-propagation in cerebellar granule cells. *J. Neurophysiol.* **101**, 519–532 (2009).
2. Hines, M. L. & Carnevale, N. T. The NEURON simulation environment. *Neural Comput* **9**, 1179–1209 (1997).
3. Cathala, L., Brickley, S., Cull-Candy, S. & Farrant, M. Maturation of EPSCs and intrinsic membrane properties enhances precision at a cerebellar synapse. *J. Neurosci.* **23**, 6074–6085 (2003).
4. Powell, K., Mathy, A., Duguid, I. & Häusser, M. Synaptic representation of locomotion in single cerebellar granule cells. *Elife* **4**, (2015).
