# Supplementary material for: Multiple signals evoked by unisensory stimulation converge onto cerebellar granule and Purkinje cells in mice
Source: Commun Biol. 2020 Jul 15;3:381. doi: 10.1038/s42003-020-1110-2 (PMC7363865; doi:10.1038/s42003-020-1110-2)
Supplement: Supplementary file 2 — Description of Additional Supplementary Files [file 42003_2020_1110_MOESM2_ESM.pdf]

Description of Additional Supplementary Files

File Name: **Supplementary Data 1**
Description: Source data for all figures
