# Supplementary material for: Multiple signals evoked by unisensory stimulation converge onto cerebellar granule and Purkinje cells in mice
Source: Commun Biol. 2020 Jul 15;3:381. doi: 10.1038/s42003-020-1110-2 (PMC7363865; doi:10.1038/s42003-020-1110-2)
Supplement: Supplementary file 4 — Reporting Summary [file 42003_2020_1110_MOESM4_ESM.pdf]

nature research

Corresponding author(s): Taro Ishikawa

Last updated by author(s): Jun 15, 2020

Reporting Summary

Nature Research wishes to improve the reproducibility of the work that we publish. This form provides structure for consistency and transparency in reporting. For further information on Nature Research policies, see our Editorial Policies and the Editorial Policy Checklist.

Statistics

For all statistical analyses, confirm that the following items are present in the figure legend, table legend, main text, or Methods section.

n/a	Confirmed	
☐	☒	The exact sample size (*n*) for each experimental group/condition, given as a discrete number and unit of measurement
☒	☐	A statement on whether measurements were taken from distinct samples or whether the same sample was measured repeatedly
☐	☒	The statistical test(s) used AND whether they are one- or two-sided<br>*Only common tests should be described solely by name; describe more complex techniques in the Methods section.*
☒	☐	A description of all covariates tested
☒	☐	A description of any assumptions or corrections, such as tests of normality and adjustment for multiple comparisons
☐	☒	A full description of the statistical parameters including central tendency (e.g. means) or other basic estimates (e.g. regression coefficient) AND variation (e.g. standard deviation) or associated estimates of uncertainty (e.g. confidence intervals)
☐	☒	For null hypothesis testing, the test statistic (e.g. *F*, *t*, *r*) with confidence intervals, effect sizes, degrees of freedom and *P* value noted<br>*Give P values as exact values whenever suitable.*
☒	☐	For Bayesian analysis, information on the choice of priors and Markov chain Monte Carlo settings
☒	☐	For hierarchical and complex designs, identification of the appropriate level for tests and full reporting of outcomes
☒	☐	Estimates of effect sizes (e.g. Cohen's *d*, Pearson's *r*), indicating how they were calculated

Our web collection on statistics for biologists contains articles on many of the points above.

Software and code

Policy information about availability of computer code

Data collection	Custom code running on Igor Pro (version 6 and 7) with NIDAQ Tools MX (WaveMetrics) is available upon request.
Data analysis	Custom code running on Igor Pro (version 6 and 7) (WaveMetrics) is available from corresponding author's website (https://sites.google.com/site/tarotoolsregister/).

For manuscripts utilizing custom algorithms or software that are central to the research but not yet described in published literature, software must be made available to editors and reviewers. We strongly encourage code deposition in a community repository (e.g. GitHub). See the Nature Research guidelines for submitting code & software for further information.

Data

Policy information about availability of data

All manuscripts must include a data availability statement. This statement should provide the following information, where applicable:

- Accession codes, unique identifiers, or web links for publicly available datasets
- A list of figures that have associated raw data
- A description of any restrictions on data availability

All data used will be made publicly available upon acceptance.

Field-specific reporting

Please select the one below that is the best fit for your research. If you are not sure, read the appropriate sections before making your selection.

☒ Life sciences ☐ Behavioural & social sciences ☐ Ecological, evolutionary & environmental sciences

For a reference copy of the document with all sections, see nature.com/documents/nr-reporting-summary-flat.pdf

Life sciences study design

All studies must disclose on these points even when the disclosure is negative.

Sample size	No statistical methods were used to predetermine sample sizes. At least n =5 and n = 6 were used for parametric and non-parametric statistical tests, respectively.
Data exclusions	All data were included unless they had poor recording quality (e.g. high access resistance in whole-cell path-clamp) as described in Methods.
Replication	No replication experiments were conducted.
Randomization	Data were not randomized because the data were compared within the same cell (or the same animal).
Blinding	At no stage were researchers blinded to sample identity.

Reporting for specific materials, systems and methods

We require information from authors about some types of materials, experimental systems and methods used in many studies. Here, indicate whether each material, system or method listed is relevant to your study. If you are not sure if a list item applies to your research, read the appropriate section before selecting a response.

Materials & experimental systems

n/a	Involved in the study	
☒	☐	Antibodies
☒	☐	Eukaryotic cell lines
☒	☐	Palaeontology and archaeology
☐	☒	Animals and other organisms
☒	☐	Human research participants
☒	☐	Clinical data
☒	☐	Dual use research of concern

Methods

n/a	Involved in the study	
☒	☐	ChIP-seq
☒	☐	Flow cytometry
☒	☐	MRI-based neuroimaging

Animals and other organisms

Policy information about studies involving animals; ARRIVE guidelines recommended for reporting animal research

Laboratory animals	VGAT-ChR2 mice, Jackson Laboratory stock no. 014548; Aldoc-Venus mice, MGI:5620954
Wild animals	The study did not involve wild animals.
Field-collected samples	The study did not involve samples collected from the fields.
Ethics oversight	All animal procedures were approved by the Institutional Animal Care and Use Committee of The Jikei University (no. 2015-054 and 2017-001) under the Guidelines for the Proper Conduct of Animal Experiments of the Science Council of Japan (2006).

Note that full information on the approval of the study protocol must also be provided in the manuscript.
